# Phytochemica: a platform to explore phytochemicals of medicinal plants

**DOI:** 10.1093/database/bav075

**Published:** 2015-08-08

**Authors:** Shivalika Pathania, Sai Mukund Ramakrishnan, Ganesh Bagler

**Affiliations:** ^1^Biotechnology Division, CSIR-Institute of Himalayan Bioresource Technology, Council of Scientific and Industrial Research, Palampur, Himachal Pradesh, India,; ^2^Centre for Biologically Inspired Systems Science, Indian Institute of Technology Jodhpur, India and; ^3^Academy of Scientific & Innovative Research (AcSIR), New Delhi, India

## Abstract

Plant-derived molecules (PDMs) are known to be a rich source of diverse scaffolds that could serve as the basis for rational drug design. Structured compilation of phytochemicals from traditional medicinal plants can facilitate prospection for novel PDMs and their analogs as therapeutic agents. *Atropa belladonna*, *Catharanthus roseus, Heliotropium indicum*, *Picrorhiza kurroa* and *Podophyllum hexandrum* are important Himalayan medicinal plants, reported to have immense therapeutic properties against various diseases. We present Phytochemica, a structured compilation of 963 PDMs from these plants, inclusive of their plant part source, chemical classification, IUPAC names, SMILES notations, physicochemical properties and 3-dimensional structures with associated references. Phytochemica is an exhaustive resource of natural molecules facilitating prospection for therapeutic molecules from medicinally important plants. It also offers refined search option to explore the neighbourhood of chemical space against ZINC database to identify analogs of natural molecules at user-defined cut-off. Availability of phytochemical structured dataset may enable their direct use in *in silico* drug discovery which will hasten the process of lead identification from natural products under proposed hypothesis, and may overcome urgent need for phytomedicines. Compilation and accessibility of indigenous phytochemicals and their derivatives can be a source of considerable advantage to research institutes as well as industries.

**Database URL**: home.iitj.ac.in/∼bagler/webservers/Phytochemica

## Introduction

Plants contain enormous number of natural compounds with important pharmacological properties, and their extracts have been used for treating various diseases from ancient times. These natural molecules have revolutionized the medicinal system (1, 2). Phytomedicines continue to play a central role in health management systems in developing countries which include 65% of the Indian population. In the USA, sale of phytomedicines has sharply increased between 1988 and 1997 (3). In Africa up to 80% of the population uses traditional medicines to help meet their health care needs. Recent World Health Organization review estimates that ∼80% of world’s population depends on traditional medicines (3).

Since Phytomedicine has globally been the matter of interest in primary source of healthcare (4) that encouraged its utilization as a source of chemical diversity in drug development. Plant-derived molecule (PDM) structures are known to have evolved under evolutionary pressure with diverse properties that make them suitable as lead structures in drug discovery (5). PDMs have also been recognized to provide specific substructures or scaffolds that make them comparable to trade drugs and their potential utilization in combinatorial chemistry (6). Such exceptional properties exhibited by PDMs make their direct use in drug discovery as well as by using them as scaffolds to synthesize combinatorial repertoire proficient enough to bind against wide range of disease-specific targets. In fact, it could be argued that plants with medicinal values may have co-evolved with humans. Various disease treatments have become dependent now upon natural products importantly diabetes (7) and cancer (6).

Besides the enormous use of PDMs and their derivatives in drug discovery there is still lack of composite repertoire of these natural molecules which can be directly used for prospection of novel leads identification. Data accessibility and its rational use have also been highlighted as important challenges to be overcome for facilitation of phytomedicines utility worldwide (3).

Application of PDMs towards lead generation and combinatorial chemistry as well as to increase their availability in rational use prompted us to compile exhaustive datasets of molecules from Himalayan bioresource. Towards our objective, we have selected few important medicinal plants such as *Atropa belladonna* (ATBE), *Catharanthus roseus* (CARS)*, Heliotropium indicum* (HEIN), *Picrorhiza kurroa* (PIKU) and *Podophyllum hexandrum* (POHX), that cover a broad range of diseases, and compiled an exhaustive set of molecules reported from literature mining. These medicinal plants have been reported in the treatment of important chronic diseases like asthma (8), Parkinson’s (9), cancer (10–12) and diabetes (13–15). Molecules of these plants have also been used to synthesize derivatives effective against cancer chemotherapy (6, 16). After a thorough literature survey, details of PDMs were manually compiled and curated to create an extensive, structured database of molecules. We present a database, Phytochemica, which is structured to include plant part source, chemical name, chemical class, IUPAC (International Union of Pure and Applied Chemistry) names, SMILES notations, and 3-dimensional (3D) structures of PDMs with all associated references. A total of 963 unique PDMs and 1854 records based on plant part source were compiled, out of which for 97 of them 3D structure could not be obtained. Physicochemical properties, including toxicity measure of all PDMs, were calculated using Discovery Studio.

**Figure 1.**
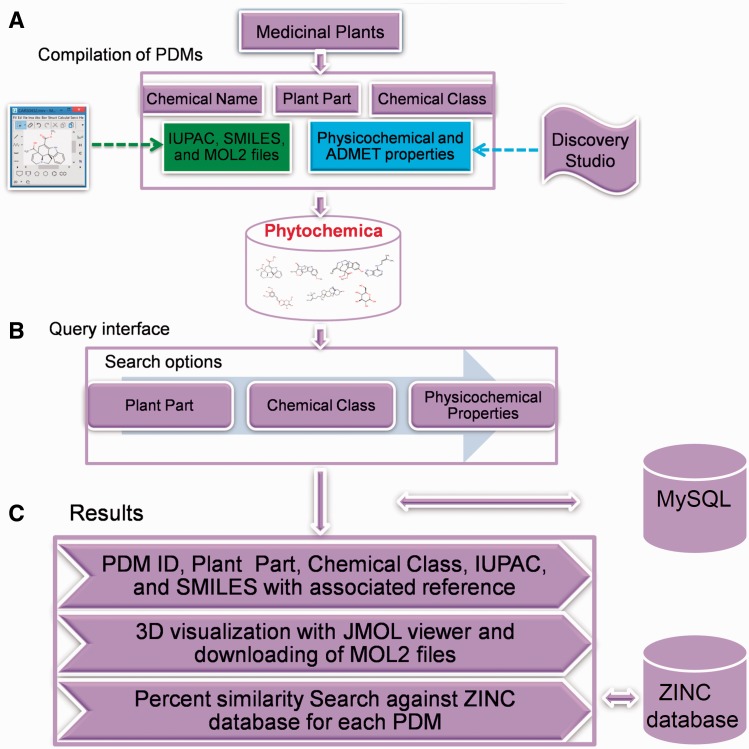
Schematic representation of overall strategy used in the Phytochemica construction.

While there are many existing repositories of natural compounds, such as NPACT (17), SuperNatural (18), Herb Ingredients’ Targets (19) and CancerResource-dataset of compound-target interactions (20), they are limited in their scope. These databases have unique features in terms of being specific to a disease, target-compound interactions, experimentally verified natural compounds etc. SuperNatural is a large repository containing 3D structures and conformers of ∼50 000 natural compounds or their derivatives. Herb Ingredients’ Targets database integrates information of 586 herbal compounds from more than 1300 well known Chinese herbs with protein-target information. While CancerResource is a cancer-specific repository that provides details of compound–target interactions, NPACT is a resource of plant-derived compounds that exhibit anti-cancerous activity. However, two of them (NPACT and CancerResource) of these are specifically focusing on cancer disease whereas Herb Ingredients’ Targets database includes only Chinese herbs. There is ample scope to include diverse bioresource, especially from Himalayan region, that are reported for their therapeutic properties. Such a repository can catalyse rational search for drugs from Himalayan medicinal plants documented for their use in traditional medicinal systems against various diseases. Phytochemica repertoire (as demonstrated in Figure 1) can facilitate prospection of novel leads and their analogs through implementation of virtual screening protocol.

To conclude, availability of phytochemical structured dataset may enable their direct use in *in* *silico* drug discovery which will hasten the process of lead identification from natural products under proposed hypothesis, and may overcome the urgent need of phytomedicines. Compilation and accessibility of indigenous phytochemicals and their derivatives can be a source of considerable advantage to research institutes as well as industries.

## Materials and Methods

### Data compilation and assembly

**Figure 2.**
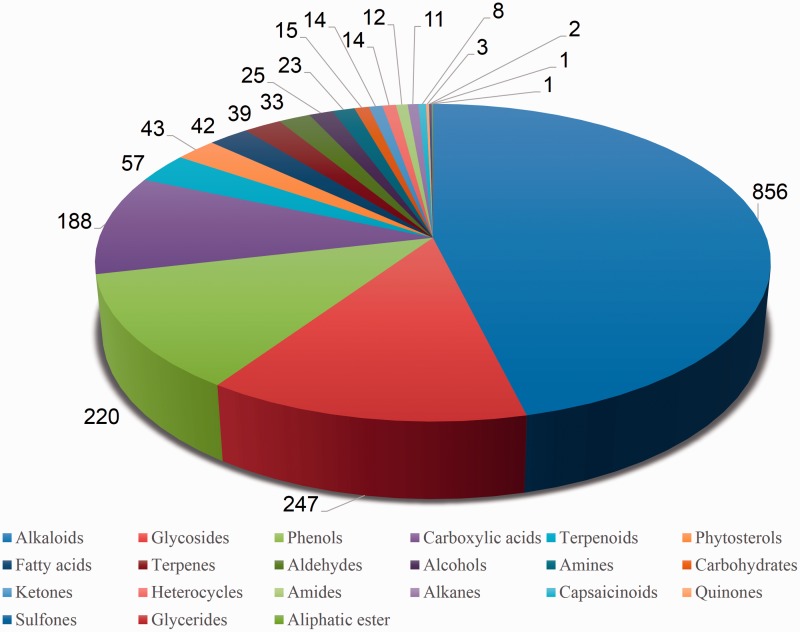
Pie chart representing phytochemical composition of PDMs within the Phytochemica.

**Figure 3.**
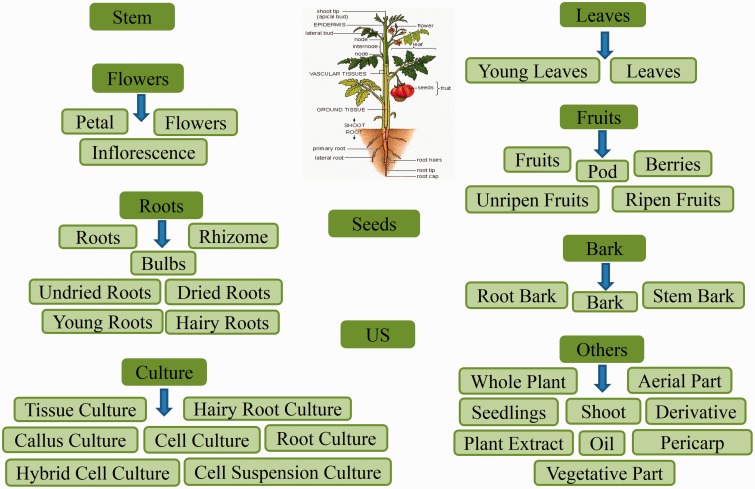
Classification schema followed to categorize the plant part source in Phytochemica. A total of 10 broad classes are obtained which further subcategorized. The PDM entry was classified as US (Unspecified) when no specific plant part, from which it was extracted, was reported.

**Figure 4.**
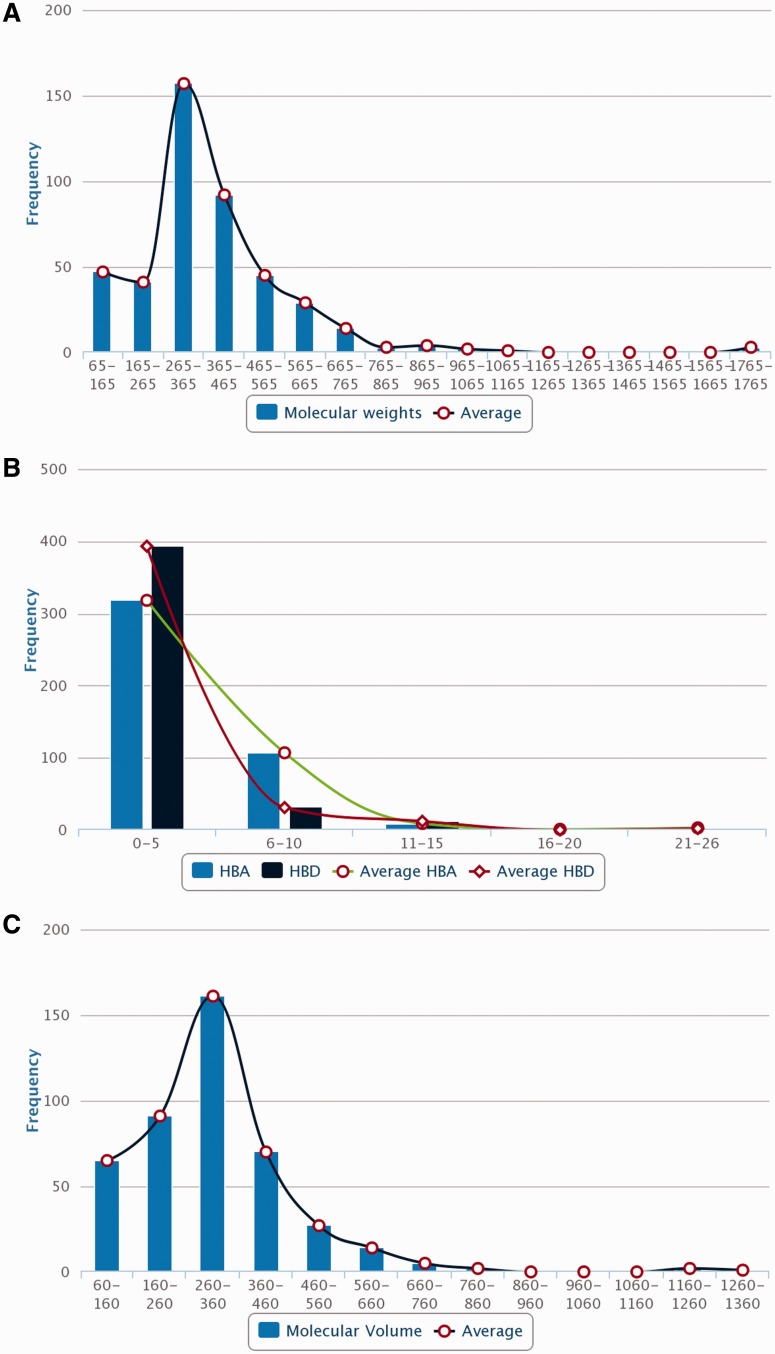
Distribution of molecular descriptors of PDMs in Phytochemica. (**A**) MW. (**B**) HBA and HBD. (**C**) MV.

In order to obtain an extensive repertoire of PDMs from these five medicinal plants (*Atropa belladonna*, ATBE; *Catharanthus roseus*, CARS; *Heliotropium indicum*, HEIN; *Picrorhiza kurroa*, PIKU; *Podophyllum hexandrum*, POHX), data were manually compiled from literature and various web resources. PubMed (http://www.ncbi.nlm.nih.gov/pubmed) was searched with plant’s scientific name and common name separately as well as along with specific keywords associated to different plant part to identify source of PDMs. Along with PubMed, various web resources were also mined to make the list more extensive and as complete as possible. A total of 281 research articles, 26 books, 15 PhD dissertations and 4 web resources (Solanaceae, http://henbane.wix.com/solanaceae; A Modern Herbal, http://botanical.com/; Obtrandon-OBat TRAdisional iNDONesia, https://obtrandon.wordpress.com/; Dictionary of Natural Products (DNP), http://dnp.chemnetbase.com/) were referred to compile an extensive list of molecules. Journals such as ‘*Phytochemistry*’, ‘*Plant medica*’, ‘*Journal of Medicinal Plant Research*’, ‘*Journal of Natural Products*’ and ‘*Journal of Pharmaceutical Sciences*’ were few of the major sources that reported these PDMs. Research articles and other resources were manually curated to archive PDMs data and their additional details including chemical name and class, plant part, IUPAC and 2D structure with all associated references. To authenticate the chemical details obtained, molecules were also ascertained from the DNP (http://dnp.chemnetbase.com/), PubChem (https://pubchem.ncbi.nlm.nih.gov/), ChemSpider (http://www.chemspider.com/) and ChEMBL (https://www.ebi.ac.uk/chembl/). Data from all the resources were merged to create a non-redundant library with a total of 963 unique PDMs (Table 1), having diverse distribution across various chemical classes (Figure 2). A separate entry was created for PDMs that were obtained from more than one plant part source leading to a total of 1854 such individual entries (Table 1). We have also included culture and derivative categories reported in the production of molecules (Figure 3). To facilitate refined prospection of molecules, we created a detailed classification schema based on plant part source as depicted in Figure 3. Further to facilitate *in* *silico* drug discovery for prospecting novel PDM leads, 3D chemical structures of PDMs were drawn and edited using MarvinSketch v5.10.0 software (https://www.chemaxon.com), an advanced chemical structure editor and saved into mol2 file format. Hydrogens were explicitly added to 2D structures to obtain 3D structures and were optimized through energy minimization, under the specifications of 500 steps of steepest descent, from Merck Molecular Force Field (MMFF94) using OpenBabel 2.3.1 software (21). The energy minimized conformers can be used as ligands directly or by searching their simpler mimetics with better pharmacological properties through virtual screening approach. A total of 866 PDMs out of 963 across five medicinal plants can be processed with their mol2 files through virtual screening protocol whereas rest 97 (ATBE, 1; CARS, 52; HEIN, 3; PIKU, 33 and POHX, 8) of them were not obtained due to unavailability of both structure as well as IUPAC. Physicochemical properties of the PDMs, such as crude energy, atomic contribution to the partition coefficient (aLogP), distribution coefficient (Logd), molecular formula, molecular mass, molecular solubility, molecular weight (MW), acid dissolution constant (pKa), number of aromatic bonds, number of aromatic rings, radius of gyration, hydrogen bond acceptor (HBA) count, hydrogen bond donor (HBD) count, number of H acceptor, number of H donor, number of H acceptor (Lipinski), number of H donor (Lipinski), number of H bonds, solvent accessible surface area, surface area and ADMET (ADMET Solubility, ADMET Solubility Level, ADMET BBB, and ADMET BBB Level) properties, were obtained using molecular property finder tool under small molecules category from the Discovery Studio v4.0 (Accelrys, San Diego, USA). Figure 4 illustrates the statistics of various physicochemical properties, such as MW (Figure 4A), HBA as well as HBD (Figure 4B) and molecular volume (MV) (Figure 4C).

**Table 1.** Total number of unique PDMs and records in Phytochemica

S. No.	Plant scientific name	Number of unique PDMs	Number of records
1	*Atropa belladonna*	85	167
2	*Catharanthus roseus*	626	1285
3	*Heliotropium indicum*	78	114
4	*Picrorhiza kurroa*	89	128
5	*Podophyllum hexandrum*	85	160
**Total**	**5**	**963**	**1854**

Difference in count is due to the multiple entries for unique PDM of different plant part.

### Database architecture and web interface

A total of four data tables were created to house the compiled data. Phytochemica implements MySQL, an object-relational database management system for its backend performance. Web browser interface was created using HTML, CSS, Ajax, JavaScript and jQuery that connects MySQL terminal using several PHP scripts. A JMol visualizer (http://www.jmol.org/) and ZINC database (http://zinc.docking.org/) has been embedded in the Graphical User Interface (GUI) to provide a 3D visualization and percentage similarity search against ZINC, respectively. The GUI is designed to be user friendly for data query and extraction, and has been tested in all major browsers (Chrome, Firefox, Safari and Internet Explorer) and OS platforms.

### Phytochemica-data access

#### Simple search

**Figure 5.**
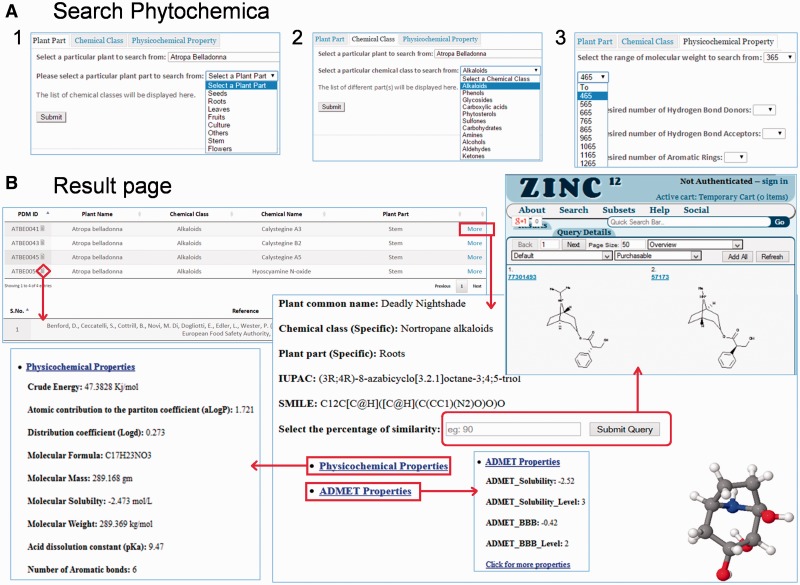
Features of Pytochemica interface. (**A**) Demonstration of accessible search options: (1) plant part, (2) Chemical class as well as plant part and (3) physicochemical properties based search of PDMs. (**B**) Result of input query with list of associated PDMs that further provides information of IUPAC, SMILES, physicochemical properties and 3D visualization with associated references. User can download mol2 file of selected PDM. Similarity search of selected PDM against ZINC is also available at user-defined cut-off.

Phytochemica can be explored for PDM data in a number of ways through querying database with a simple text search tool that provides various options for searching. There are three search sections available to the user with several constraints in each. Search can be performed for specific plant along with (i) plant part, (ii) chemical class and (iii) physicochemical properties (Figure 5A). Physicochemical properties search option has advanced the search query options for user to select PDMs in a particular range based on MW, number of HBA or donor, XLogP, polar surface area and inhibitory concentration. The result page for a given query is demonstrated in a new page (Figure 5B) which is represented with information such as PDM ID, plant part, chemical name, chemical class, IUPAC name, SMILES notation and 3D structure of PDM with associated references. A total of 20 PDM entries are displayed on single page and ordered by PDM ID that can be reordered later according to other fields as well. While clicking a PDM ID, the information of physicochemical properties like 1D, 2D, 3D and ADMET are presented in other window. Also, a JMol visualizer (http://www.jmol.org/) has been embedded in the GUI to provide a 3D visualization of PDM which can be downloaded to mol2 file format.

#### Percentage similarity search

Each resulted PDM can be searched further for similar compounds at different percentage of similarity cut-off (default 90%). In order to perform this search ZINC database, a curated collection of commercially available chemical compounds (22), is hyperlinked to result page for each PDM separately and return their structural analogs with a user defined cut-off. During this similarity search natural molecules are used as scaffolds to search for similar mimetics by searching the neighbourhood of chemical space. Thus, phytochemica serves as a portal to facilitate the impact of natural chemical diversity on drug discovery through prospection of direct novel leads and their analogs.

## Discussion

Phytochemica provides comprehensive information of PDMs from five medicinal plants (*A*. *belladonna*, *C*. *roseus, H*. *indicum*, *P*. *kurroa*, *P*. *hexandrum*) as a structured and integrated library. This database was developed to facilitate prospection of therapeutic molecules from these medicinally important plants. Existing repositories of natural compounds, such as NPACT, SuperNatural, Herb Ingredients’ Targets and CamMedNP, focus on different utilitarian aspects of PDM libraries. Although some of these databases emphasize on a specific disease or target-compound interactions, others cover plants of specific geography. Phytochemica contains natural molecules of which is an important Himalayan medicinal plant having various therapeutic properties reported to cover broad range of chronic diseases. This database can facilitate prospection of novel leads and their analogs for various diseases from the repertoire of natural molecules.

Large-scale deforestation and over exploitation of medicinal plants for therapeutic molecules, which specifically depends upon the underground part for their extraction, further reduces their number. Plant regeneration through micropropagation techniques have been used to conserve biodiversity and to overcome present demand of such medicinal plants (23). Since Phytochemica has separate entry on the basis of different plant part source (Figure 3) for given PDM. Therefore, information of culture techniques, mentioned as plant part source, is beneficial for researchers who deal with the regeneration of such endangered medicinal plants for the controlled synthesis of natural molecules.

Natural molecules have been recognized to provide specific scaffolds that make them comparable to trade drugs and their potential utilization in combinatorial chemistry (24). The MW distribution of PDMs present in Phytochemica has been found to follow Gaussian distribution and peaked in the range of 265–365 Da (Figure 4A) which is similar to drug-like molecules of previously reported libraries of natural products (25). Significant numbers of PDMs have HBA in the range of 4–5 with a sharp decline thereafter, as desired from drug-like molecules (Figure 4B). Similarly, HBDs of PDMs have a peak at 2–3 with a sharp drop for higher values, as desired (Figure 4B). Phytochemica PDMs have maximum density in the ‘Lipinski region of interest’ reflecting their drug-like properties and hence, their utility in prospection of novel leads. The relevance of natural molecules in drug discovery has been demonstrated with the virtual screening protocol, molecular dynamics, and ZINC similarity search for potential inhibitors of aldose reductase, a target for complications of diabetes (14). This hypothesis driven prospection study yielded two indole alkaloids as well as their structural analogs as potential AR inhibitors (14). This protocol serves as a demonstration of utility natural molecules and their analogs for rational search of therapeutic molecules that further highlights its relevance. Future extensions of Phytochemica may include 3D structure similarity search and disease associations for each PDM.
